# Somatostatin Treatment for Ectopic ACTH Syndrome due to Pancreatic Neuroendocrine Tumors: Review of the Literature

**DOI:** 10.1155/2022/6283706

**Published:** 2022-02-28

**Authors:** Da Zhang, Lin Lu, Hui-Juan Zhu, Yu Xiao, Xian-Lin Han, Shun-Da Du, Hua-Dan Xue, Qing-Xing Liu, Zhao-Hui Zhu, Ming-Ming Hu, Xiao Zhai, Xiao-Ping Xing, Zhao-Lin Lu

**Affiliations:** ^1^Department of Endocrinology, Air Force Medical Center, People's Liberation Army, Beijing 100142, China; ^2^Department of Endocrinology, Key Laboratory of Endocrinology of National Health Commission, Peking Union Medical College Hospital, Chinese Academy of Medical Science and Peking Union Medical College, Beijing 100730, China; ^3^Department of Pathology, Peking Union Medical College Hospital, Chinese Academy of Medical Science and Peking Union Medical College, Beijing 100730, China; ^4^Department of General Surgery, Peking Union Medical College Hospital, Chinese Academy of Medical Science and Peking Union Medical College, Beijing 100730, China; ^5^Department of Radiology, Peking Union Medical College Hospital, Chinese Academy of Medical Science and Peking Union Medical College, Beijing 100730, China; ^6^Department of Nuclear Medicine, Peking Union Medical College Hospital, Chinese Academy of Medical Science and Peking Union Medical College, Beijing 100730, China

## Abstract

**Objectives:**

To analyze and summarize the effect of SSA treatment on EAS due to p-NETs (EAS-p-NETs).

**Methods:**

Thirteen patients with EAS-p-NETs treated with SSAs at our center or described in the literature were included in this study. Clinical characteristics, laboratory data, imaging studies, histopathologic results, the effect of SSA treatment, and the prognosis of these EAS-p-NET patients were evaluated.

**Results:**

Four males and 9 females with an average age of 42.9 years were included in the study. The mean duration of follow-up was 38.8 ± 28.2 months. As one of the combined treatment measures, SSAs controlled the levels of ACTH and cortisol in 9 of the 13 patients (69.2%). Partial response was observed in 3 patients (23.1%), stable disease in 2 patients (15.4%), and progressive disease in 6 patients (46.2%). The median time to tumor progression was 24 months, and the median overall survival was 61 months. The side effects of SSA treatment included temporary mild abdominal pain, diarrhea, gallstones, and cholecystitis.

**Conclusions:**

As a supplemental therapy, SSA treatment led to clinical and biochemical improvement with a good safety profile in patients exhibiting EAS-p-NET with metastasis. However, tumor progression was inhibited by SSA treatment in only a few patients. Combined with other treatments, SSAs may improve the prognosis of patients with EAS-p-NETs.

## 1. Introduction

Cushing's syndrome (CS) is a rare endocrine disease. The prevalence of CS ranges from 0.7 to 2.4 per million people per year [1]. CS is classified into adrenocorticotropic hormone (ACTH)-dependent and ACTH-independent causes. Approximately 10–20% of CS cases are due to ectopic ACTH syndrome (EAS), which originates from an ACTH-producing or corticotropin-releasing hormone (CRH)-producing neuroendocrine tumor outside of the pituitary [2]. EAS has been reported to be due to small-cell carcinoma of the lung, bronchial carcinoid tumors, thymus carcinoid, pheochromocytoma, pancreatic neuroendocrine tumors (p-NETs), and gut carcinoids. p-NETs are present in up to 16% of EAS cases [3]. p-NETs are a group of heterogeneous diseases with varying tumor biology and clinical presentations. Unfortunately, p-NETs often metastasize widely by the time the clinical manifestations of CS appear. The overall 5-year survival rate of patients with high-grade p-NETs does not exceed 40–60% [4, 5].

The treatment of EAS from p-NETs with metastasis is quite challenging. The first goal is to control the excess production of ACTH or CRH and relevant clinical symptoms of hypercortisolism. Another goal is to control tumor progression. Somatostatin analogs (SSAs) are the first-line therapy for functional p-NETs, such as unresectable insulinoma, vasoactive intestinal polypeptide secreting tumors (VIPomas), and glucagonoma [6]. A meta-analysis systematically showed an antiproliferative effect of SSAs in advanced gastroenteropancreatic neuroendocrine tumors (GEP-NETs) [7]. In the management of GEP-NETs, stabilization of tumor growth, rather than tumor shrinkage, is the most frequent response to treatment. SSAs stabilize tumor growth in 36–70% of patients with metastatic GEP-NETs [8]. The rate of tumor progression in patients with pancreatic primary sites in the CLARINET study was 42.8% in the SSA group and 63.3% in the placebo group [9]. However, EAS is a rare manifestation in functional p-NETs. At present, there are few studies regarding the effect of SSAs in EAS due to p-NETs, and large-scale research is lacking. Thus, the effect of SSA treatment on EAS due to p-NETs should be evaluated.

In this article, we present a case of ectopic CRH syndrome stemming from a p-NET with liver metastasis successfully treated with octreotide long-acting release (LAR) and analyze the clinical characteristics and prognosis of 12 patients with EAS due to p-NETs treated with SSA in the literature to analyze and summarize the effect of SSA treatment on EAS due to p-NETs.

## 2. Methods

### 2.1. Medical Information of This Case

The medical records of the patient with an EAS-p-NET with multiple liver metastases at our hospital were reviewed. Information related to clinical characteristics, laboratory data, imaging studies, histopathologic results, treatment, and prognosis was collected. This study was approved by the Institutional Review Board of our hospital. Informed consent was obtained from the patient and her parents.

CS was diagnosed according to the 2008 clinical practice guidelines for the diagnosis of CS [10]. A level of morning ACTH persistently greater than 15 pg/mL almost always results from ACTH-dependent etiology [11]. Bilateral inferior petrosal sinus sampling (BIPSS) is recommended in cases with no detection of a visible pituitary adenoma on MRI, no suppressible high-dose dexamethasone suppression test (HDDST), and suspected ectopic lesions to differentiate the causes. A basal central : peripheral ratio of ACTH greater than 2 : 1 at baseline and/or greater than 3 : 1 after stimulation with CRH or desmopressin (DDAVP) is consistent with Cushing's disease, otherwise indicating a diagnosis of EAS [12]. Subsequently, chest CT, abdominal CT, octreotide scanning, and PET/CT are carried out to detect the ectopic lesions individually.

The antitumor response to SSAs was assessed every 2-3 months using CT or MRI. The tumor response was graded according to the Response Evaluation Criteria in Solid Tumors (RECIST) version 1.1 [13]. Complete response (CR) refers to the disappearance of all target lesions. Partial response (PR) refers to at least a 30% decrease in the sum of the diameters of the target lesions. Progressive disease (PD) refers to at least a 20% increase in the sum of the diameters of the target lesions or the appearance of one or more new lesions. Stable disease (SD) refers to neither sufficient shrinkage to qualify for PR nor sufficient increase to qualify for PD. The median time to tumor progression (TTP) and median overall survival (OS) were calculated using the Kaplan–Meier method.

### 2.2. Literature Review

A systematic literature review was conducted by searching PubMed, Web of Science, and EMBASE for all relevant and available articles published in English. MeSH terms included “somatostatin/administration and dosage/analogs and derivatives/therapeutic use,” “octreotide/administration and dosage/analogs and derivatives/therapeutic use,” “ACTH syndrome, ectopic,” “neuroendocrine tumors,” and “pancreas” and “pancreatic.” Original research, case reports, case series, or review articles published between 1950 and 2020 were included. Studies analyzing cases that were without somatostatin treatment or were not from p-NETs were all excluded.

### 2.3. Whole-Exome Sequencing of the Blood and Tumor Tissue

Whole-exome sequencing (WES) was performed using germline DNA samples and tumor DNA samples from our patient. DNA of peripheral blood leucocytes was captured using an Agilent SureSelect Human All Exon v6 (58 Mb) Kit (Agilent Technologies) and sequenced on an Illumina HiSeq 2000 (Illumina) with 150 bp paired-end reads. Sequence reads were mapped to the hg19/GRCh37 human reference genome. Alignment and variant calling were performed using the Burrows–Wheeler Aligner (BWA)-MEM and the Genome Analysis Toolkit (GATK V.3.7.0), respectively, with default parameters. The number of total reads generated was 109 million, resulting in a median target coverage of 145 × (97.64% of targets covered at >20x). For USP8, the median percent of bases covered at 20x was 100% for both coding exons. The output variant call format (VCF) files were annotated to identify and classify the disease-relevant variants using ANNOVAR software (https://www.openbioinformatics.org/annovar/) and ENSEMBL Variant Effect Predictor v91. All identified variants were annotated according to the guidelines published by the Human Genome Variation Society (HGVS). Bioinformatic analyses were used to rule out rare variants (present in <0.5% of allele frequency) in known p-NET-associated genes (e.g., AIP, GNAS, PDE8B, MEN1, PRKAR1A, USP8, and SDHx). Targeted amplicon sequencing using DNA derived from peripheral blood leucocytes and Sanger sequencing using a 3730XL autosequencer (Applied Biosystems) were used to confirm mutations identified by WES. Sequences were analyzed using SeqMan 7.0 (DNASTAR) software.

## 3. Results

### 3.1. Case Presentation

A 23-year-old female patient presented in April 2018 with a history of emotional changes, insomnia, increased appetite, weight gain, acne, fatigue (especially when climbing stairs), and irregular menstruation for 3 months, and she gradually developed Cushingoid manifestations with edema of the bilateral lower extremity and generalized fatigue. Initial laboratory evaluation revealed hypokalemia (3.3 mmol/L, normal range 3.5–5.5), hyperlipidemia, and impaired liver function. Her condition was also complicated with new onset diabetes and hypertension.

**Figure 1:**
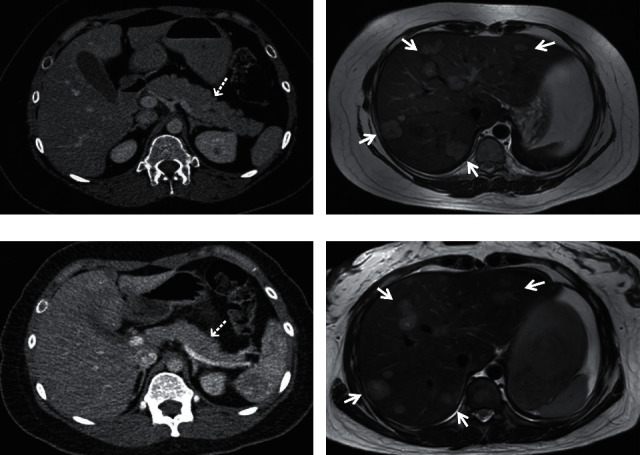
Abdominal CT and MRI images before (a and c) and after 7 cycles of octreotide LAR therapy (b and d) (case 1). (a) The abdominal CT image demonstrated a solid nodule in the body-tail junction of the pancreas (dotted arrow). (b) The abdominal MRI demonstrated multiple blood-rich nodules in the liver (arrow). (c) The abdominal CT image demonstrated that the pancreatic solid nodule was unclear after 7 cycles of octreotide LAR therapy (dotted arrow). (d) The abdominal MRI demonstrated that the sizes and numbers of hepatic metastatic nodules remained stable (arrow). CT: computed tomography. MRI: magnetic resonance imaging.

**Figure 2:**
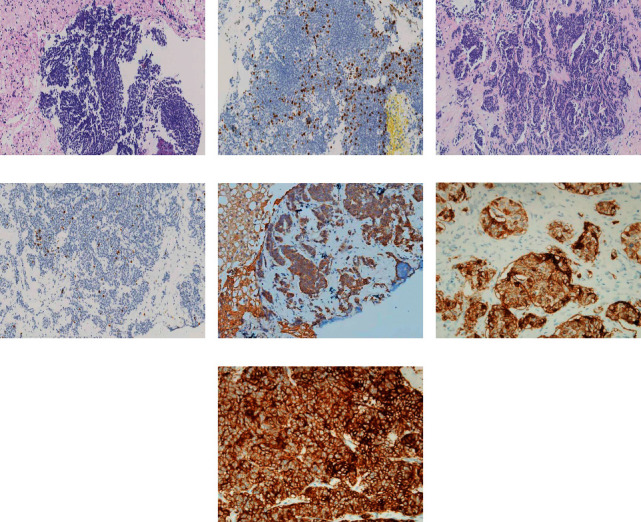
Pathology and immunochemistry staining of tumors of the pancreas (a, b, e, and f) and liver metastasis (c, d, and g). (a) Pancreatic neuroendocrine tumor (hematoxylin and eosin staining, magnification ×200); (b) the percentage of Ki-67-positive cells (brown color) was 15% in the pancreatic tumor (magnification ×200); (c) liver metastases of the neuroendocrine tumor (hematoxylin and eosin staining, magnification ×200); (d) the percentage of Ki-67-positive cells (brown color) was 10% in the hepatic metastases (magnification ×200); (e) positive immunostaining for CRH (brown color) in the liver metastasis (magnification ×200); (f) & (g) positive immunostaining for SSTR2 (brown color) in the pancreatic tumor (magnification ×400) (f) and in the hepatic metastases (magnification ×400) (g).

Endocrine workup showed that the morning serum cortisol level increased to 24.10 *μ*g/dL (reference range: 4.0–22.3 *μ*g/dL), the midnight cortisol level increased to 35.23 *μ*g/dL, and the overnight low-dose (1 mg) dexamethasone suppression test was not suppressed. Elevated 24 h urinary free cortisol (UFC) excretion (653.64 *μ*g per day; reference range: 12.3–103.5) was found. The morning plasma ACTH level was significantly increased to 139.0 pg/mL (reference range: 0–46 pg/mL). No central-to-peripheral ACTH gradient was observed on BIPSS (the ratio of central/peripheral ACTH was 1.12 at baseline and 1.20 after DDAVP stimulation). Abdomen high-resolution CT/MRI demonstrated multiple blood-rich nodules in the liver and a solid nodule (1.1 × 0.8 cm) in the body-tail junction of the pancreas, with mild hyperplasia of adrenals (Figure 1). Somatostatin receptor scintigraphy (SRS) and 18F-FDG-PET/CT revealed multiple metastatic foci of high uptake in the liver with a standardized uptake value (SUV) of 4.2–6.6 but no uptake of pancreatic lesions. ^68^Ga-DOTATOC PET/CT was performed further, showing increased uptake in both metastatic nodules in the liver and primary pancreas. The maximum SUV of the pancreatic tumor was 20.7 and that of the liver metastases was 21.4–26.6. Histological examination after biopsy demonstrated the presence of a p-NET and liver metastases with positive immunostaining for chromogranin A and synaptophysin. The pancreatic tumor and liver metastases had a Ki-67 index of 15% and 10%, respectively. Interestingly, the tumors showed positive immunohistochemical staining for CRH but negative staining for ACTH (Figure 2).

Short-term (13 days) octreotide therapy (100 *μ*g subcutaneously, q8h) prompted marked reductions in the ACTH level from 131 to 63 pg/mL and in the cortisol level from 57.47 to 7.16 *μ*g/dL, and the UFC level decreased to the normal range (55 *μ*g per day). The recommended treatment for this patient was octreotide LAR at a dose of 20 mg every 28 days for 7 cycles, leading to a favorable clinical and biochemical response. The hormonal tests were maintained in the normal range with the ACTH level ranging between 23.0 and 53.4 ng/L and the UFC level ranging between 6.96 and 48.84 *μ*g/24 h. During the first two weeks, the patient experienced transient mild abdominal pain and diarrhea. After 7 cycles of somatostatin LAR treatment, abdominal MRI showed that the primary and metastatic tumors remained stable. However, the enhanced intensity of the lesions was reduced on contrast MRI, indicating that the blood supply of the lesions had decreased (Figure 1). ^18^F-FDG PET-CT also demonstrated slightly decreased radioactivity uptake in the liver metastases, with SUVmax values of 3.5–6.0, and ^68^Ga-DOTATOC PET-CT revealed that the SUVmax in the liver foci decreased to 17.1–20.2, while the SUVmax in the pancreatic foci decreased to 11.6.

**Figure 3:**
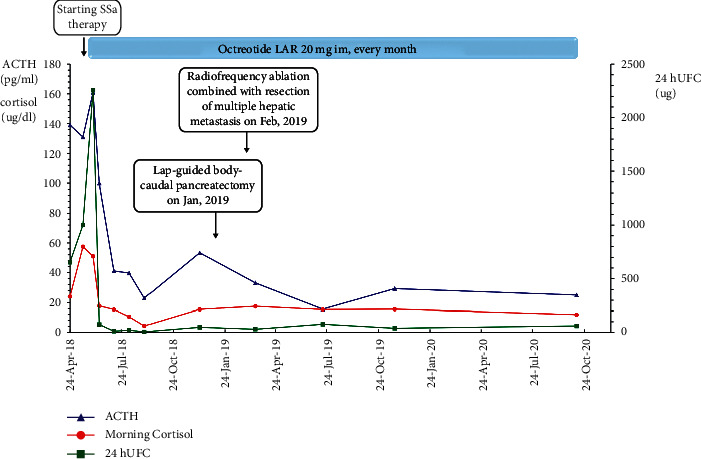
ACTH, morning cortisol, and UFC trends during the follow-up after SSA treatment and surgery. Short-term (13 days) octreotide therapy prompted marked reductions in ACTH levels (from 131 to 63 pg/mL) and cortisol levels (from 57.47 to 7.16 *μ*g/dL), and UFC levels decreased to the normal range (55 *μ*g per day). Surgery and long-term octreotide LAR therapy maintained the hormones normally, with ACTH fluctuating from 23.0 to 53.4 ng/L and UFC fluctuating from 6.96 to 48.84 *μ*g/24 h.

The patient underwent laparoscopic-guided body-caudal pancreatectomy plus splenectomy after the 8th cycle injection of octreotide LAR. Radiofrequency ablation combined with resection of multiple hepatic metastases was conducted 3 weeks later. The Ki-67% indices of the pancreatic tumor and liver metastases were 10% and 4%, respectively. Immunostaining of somatostatin receptor 2 (SSTR2) was carried out to show positive results. Octreotide LAR treatment was continued for 24 cycles, and the patient received regular follow-up, showing long-term normalization of the levels of ACTH, cortisol, and 24 h UFC (Figure 3). The only side effect was mud-like gallstones suggested by ultrasound, without any symptoms. In October 2020, abdominal MRI and ^68^Ga-DOTATOC PET/CT revealed some new liver metastatic lesions, with an SUVmax of 11.5. The patient underwent peptide receptor radionuclide therapy (PRRT) 6 times, once every 3 months, and received chemotherapy with capecitabine and temozolomide starting in October 2020. The liver metastatic foci diminished after PPRT and chemotherapy.

### 3.2. Literature Review

Twelve references were included that involved patients with EAS due to p-NETs, and all the references were published as case reports. There were 12 patients (4 male and 8 female) with EAS originating from p-NETs who were treated with SSA as one of the therapeutic strategies described in these reports (Table 1). The mean age at diagnosis was 42.9 ± 13.2 years. All the patients had typical Cushingoid manifestations. Ten of the 12 patients had hypokalemia with a mean value of 2.56 ± 0.57 mmol/L (range from 1.7 to 3.8 mmol/L). Ten patients were complicated with diabetes, and 8 patients had hypertension. The mean morning ACTH level was 269.6 ± 183.8 pg/mL (range from 68.1 to 735 ng/L). The median morning cortisol and UFC levels were 40 *μ*g/dL (range from 15.2 to 1198 *μ*g/dL) and 986.5 *μ*g/day (range from 86.1 to 38,200 *μ*g/day), respectively. The size of the primary pancreatic tumor ranged from 1.2 to 6.0 cm (3.7 ± 2.0 cm). All the patients had metastasis at diagnosis. Five of the 12 patients had lymph node metastases, and 10 of the 12 patients had liver metastases. The median Ki-67 index of the pancreatic or metastatic tumors was 10.7% (<1% to 20%). Immunostaining for SSTR2 was performed in 2 patients, both of which were positive. Immunostaining for SSTR5 in liver metastatic lesions was performed in 1 patient, and the tumor was positively stained.

### 3.3. SSA Effects in Patients with EAS due to p-NETs

We evaluated our reported case together with the 12 cases from the literature review to analyze the effect of SSA in controlling hormone production and tumor growth.

#### 3.3.1. The Effect of SSA on the Excess Production of Hormones

The effect of controlling hypercortisolism after SSA treatment was recorded in 12 patients except patient 4. ACTH and cortisol levels decreased to normal ranges after SSA therapy alone in 8 of those 12 patients (66.7%). Combined with metyrapone, SSA effectively decreased the ACTH and cortisol levels in 1 patient. The types of SSA included octreotide LAR (20 mg per month), lanreotide, and octreotide (50 *μ*g/d to 1500 *μ*g/d). ACTH and cortisol were normalized in 8 patients who exhibited a good response in the octreotide loading test. The effect of decreasing the ACTH and cortisol levels appeared a few days after the onset of SSA application and was sustained for an average of 11.2 months (range: 2–24 months).

#### 3.3.2. The Effect of SSA on Tumor Growth and Prognosis

The effect on tumor growth after SSA treatment was available in 11 patients with a mean follow-up time of 38.8 ± 28.2 months. Primary or metastatic tumor sizes decreased in 3 patients (27.3%), remained stable in 2 patients (18.2%), and progressed in size or number in 6 patients (54.5%). The prognosis was mentioned for 7 patients. The median TTP was 24 months, and the median OS was 61 months. Four patients died of tumor progression or complications, including intestinal perforation, ARDS, and severe hepatic insufficiency. One patient remained clinically stable after chemotherapy, another patient planned to undergo radical surgical treatment, and our patient underwent PRRT.

#### 3.3.3. Side Effect of SSA Treatment

The side effects of SSA were available for 7 patients. No side effects were recorded for 3 patients. Temporary mild abdominal pain, diarrhea, gallstones, and cholecystitis occurred in 4 patients. Additional glucocorticoid supplementation was not reported for any of the patients, even those in whom the ACTH and cortisol levels decreased rapidly. There were no treatment delays or interruptions due to adverse events.

## 4. Results of WES

In the present case, WES of the patient's peripheral blood and tumor tissue was carried out to investigate any genetic changes. A heterozygous variant of the *USP8* gene (p.P750A) inherited from her mother with unclear clinical significance was detected. Other related abnormalities were absent.

## 5. Discussion

Functional p-NETs are rare, representing approximately 1% of pancreatic neoplasia [26]. Ectopic ACTH-producing tumors are very rare types of functional p-NETs, with few reports in the literature and no consensus guidelines for management. Retrospective studies support the use of octreotide LAR in functioning and nonfunctioning low-grade p-NETs but not in p-NETs secreting ACTH or CRH. Hence, this study analyzed and summarized the effect of SSA treatment on EAS due to p-NETs to provide evidence for this rare situation.

The inhibited secretion of peptides from the NET by SSA is mediated mainly through SSTR2 and SSTR5. SSTR2 is expressed in almost 80% of p-NETs [27]. SRS and ^68^Ga-DOTATOC PET-CT can provide not only a better overall detection rate for diagnosis but also indications regarding SSTR expression on target lesions, allowing a decision regarding SSA treatment [28]. In addition, an in vivo octreotide loading test or immunostaining with SSTR2 and SSTR5 in tumor tissues may help to further predict the therapeutic response of SSA [29]. The positive octreotide loading test predicted the good effect of SSA in 7 patients in this case series. However, negative SRS or ^68^Ga-DOTATOC PET-CT could not determine whether there was no effect of octreotide treatment. Initiating therapy with octreotide 50–100 *μ*g s.c. for 7–10 days twice to thrice per day is recommended for gastroenteric NETs [30].

Although the expression of SSTR2 and SSTR5 has predictive value for SSA effects, notably, not all p-NET tissues from a different focus in a patient express similar subtypes of SSTRs. Therefore, each of the metastatic tumors might have a differential expression of SSTR subtypes [19]. The heterogeneity and pluripotency of p-NETs explain why some tumors respond well to SSAs while others either show no response or become resistant to treatment within months. In our study, the expression of SSTR2 or SSTR5 was detected in cases 1, 7, and 8, in which SSA had good effects on the control of hypercortisolism. The longest period of symptom control was 28 months. The potential mechanisms of nonresponsiveness may include downregulation of SSTRs or the outgrowth of clones lacking specific SSTR expression [31].

SSAs are generally recommended as a first-line treatment to slow disease progression in patients with advanced p-NETs by the North American Neuroendocrine Tumor Society because of the lack of toxicity of these drugs and the fact that they are well tolerated [32]. In a review of 15 studies including 481 patients with functional GEP-NETs, octreotide LAR achieved symptomatic relief in 74.2% of the patients and a biochemical response in 51.4% [33]. EAS-p-NETs had a total control rate of 75% in terms of symptomatic and biochemical control under first-line therapy with an SSA. The side effects of the SSA were mild [34]. However, some patients may develop gallstones after long-term treatment. According to our data, SSAs may be chosen as the first-line preoperative therapy in patients with EAS-p-NETs.

Apart from symptom control, SSAs also have the potential to control tumor growth. Among all 13 patients with EAS-p-NETs, tumor progression was controlled in 5 (38.5%). Three patients (23.1%) exhibited tumor progression during SSA therapy, although ACTH production was well controlled by SSA treatment. The median TTP for Ki-67 > 10% was 4 months, that for Ki-67 < 5% was 15 months, and that for Ki-67 5–10% was 12 months [35]. Octreotide treatment has been reported to stabilize tumor growth in 36.5% of metastatic endocrine GEP-NETs for at least 12 months without causing tumor regression [36]. PR and SD of tumor growth have been demonstrated in up to 10% and approximately 50% of functional GEP-NET patients [37]. In one Korean study, PR and SD were achieved in 2.2% and 88.9% of p-NET patients, respectively. The median TTP was 16.4 months (95% confidence interval, 9.5–23.3 months) [34]. Jann et al [35] retrospectively analyzed the records of 43 patients with p-NETs (19 functional tumors without EAS-p-NETs, 24 nonfunctional tumors) treated with octreotide LAR. After 12 months of SSA therapy, there was a total disease control rate of 42%, including PR in 4.7% and SD in 37.2% of patients when evaluating tumor growth. The median OS for SSA responders (SD + PR) was 137 months versus 68 months for nonresponders (PD). The median TTP was 22 months for SSA responders and 3 months for nonresponders. Our data indicated that PR was achieved in 23.1% of the patients (3/13) and that SD was achieved in 15.4% of the patients (2/13), with a median TTP of 24 months and a median OS of 61 months in patients with EAS-p-NETs. The antiproliferative effect of SSAs exhibited a disparity in different studies of GEP-NETs. However, previous studies have not evaluated the efficacy of SSAs in controlling tumor growth in EAS-p-NETs. In this study, the relatively low rate of tumor proliferation control by SSA may have been found because 66.7% of the patients had pathological features with Ki-67% ≥ 10% at baseline, which might have a poor response to SSA, as in previous reports.

Considering the prognosis, grading of NETs, distant metastases, and severity of hypercortisolism may affect the prognosis of EAS patients [38]. The five-year survival rate was 78% without and 27% with distant metastases [39]. In our series, all the EAS-p-NET patients had distant metastases at the time of diagnosis. A multidisciplinary approach should be adopted to improve the prognosis of patients. Surgery with curative intent always has to be considered in grade 1 and 2 NETs, even if liver metastases are present [40]. P-NET patients with distant metastatic disease who underwent surgical resection of the primary tumor showed significantly better survival [39]. Patients with EAS who underwent surgery had a better survival rate than those who did not (5-year OS 85% vs. 51%, *P* < 0.001) [38]. Successful control of hypercortisolemia with SSAs provides an opportunity for further surgery and markedly reduces the operative and perioperative risk since EAS patients often have a high risk of fatal opportunistic infection, severe hypopotassemia, hyperglycemia, and hypertension. Thus, resection of the primary tumor and debulking surgery for metastases should be considered after controlling hypercortisolemia by SSA to improve the prognosis of EAS from p-NETs.

There are some limitations in this study. Initially, this was a retrospective study with a small sample size, as EAS-p-NETs are quite rare, and the effect of tentative treatment with an SSA may be variable. As follow-up in most cases was rather short, the duration of the possible antiproliferative action of SSAs is unknown. In addition, not all patients underwent immunostaining of SSTRs, octreotide loading tests, or radiolabeled molecular imaging tests. Future research is required to assess the long-term efficacy of SSAs and improve the prognosis of EAS-p-NETs.

In summary, EAS-p-NETs are rare endocrinological entities associated with severe hypercortisolism and are difficult to manage since metastasis is common at diagnosis. Diagnostic imaging with SRS and 68 Ga-DOTATOC PET-CT and immunostaining with SSTR2 provide information on SSTR expression status in the tumor, which combined with an octreotide loading test helps to predict the response to SSA treatment. SSAs may be used as first-line therapy before surgery and can lead to both instant clinical improvement with a good safety profile and control of tumor growth in some patients with EAS-p-NETs. SSAs could ameliorate severe conditions in patients and give them the chance to undergo subsequent surgery to improve their prognosis.

## Figures and Tables

**Table 1:** Clinical characteristics of 13 patients with EAS-p-NETs.

Year	First author	Age at diagnosis (year)	Sex	Morning ACTH (pg/ml)	Morning cortisol (ug/dl)	24 h UFC (ug/d)	Pancreatic tumor size (cm)	Tumor metastasis	Percentage of Ki-67	Treatment procedure	Effect of controlling ACTH and cortisol after SSA treatment	Effect of tumor growth after SSA treatment	Duration of follow-up (months)	Prognosis
2020	Our case	23	F	139	24.1	653.64	1.1 × 0.8	Lymph nodes and multiple liver metastases	10–15%	Octreotide LAR and operation	Controlled	**SD**	28	Survival
2017	do Amor Divino et al. [14]	58	M	190	48	6519	5.0	Multiple liver metastases	6%	Operation, octreotide LAR and chemotherapy	Uncontrolled	**PD**	42	N/A
2016	Tadokoro et al. [15]	61	F	205.9	15.2	86.1	6.0	Lymph nodes metastasis	11.50%	Octreotide LAR and chemotherapy	Uncontrolled	**PD**	36	Died
2014	Sauer et al. [16]	46	M	247	1198	38200	1.2	Liver metastasis	<1%	Octreotide and operation	N/A	N/A	N/A	N/A
2014	Rajeev et al. [17]	34	M	227	72.6	N/A	N/A	Lymph nodes and liver metastasis	20%	Operation, lanreotide, and sunitinib	Uncontrolled	**PD**	27	Survival
2013	Patel et al. [18]	44	F	N/A	N/A	N/A	Large	Multiple liver metastases	N/A	Operation and octreotide LAR	Controlled	N/A	74	N/A
2010	Kondo et al. [19]	64	F	340	26.9	893	2.5 × 2.5	Multiple liver metastases	10%	Octreotide LAR and operation	Controlled	**PR**	20	Survival
2003	Doi et al. [20]	21	F	735	145	N/A	5.0 × 3.5 × 2.0	Liver and lymph nodes metastasis	N/A	Octreotide and operation	Controlled	**PD**	61	Died
1999	Gill et al. [21]	31	F	177	78.2	2102	N/A	Liver, bone, and pelvic metastasis	N/A	Octreotide and operation	Controlled	**PD**	18	Died
1993	Woodhouse et al. [22]	33	F	436	181.2	N/A	N/A	Extensive liver metastasis	N/A	Operation and octreotide	Controlled	**PR**	18	N/A
1989	Bertagna et al. [23]	37	F	218	26	550	N/A	Diffuse metastasis	N/A	Chemotherapy and octreotide	Controlled	**PD**	4	Died
1988	Lamberts et al. [24]	37	M	68.1	32	1080	5.0 × 3.0	Lymph nodes metastasis	N/A	SMS 201-995	Controlled	**PR**	6	N/A
1988	Ruszniewski et al. [25]	49	F	138	16.5	N/A	N/A	Multiple liver metastases	N/A	Operation, chemotherapy, and SMS 201-995	Controlled	**SD**	80	N/A

EAS: ectopic ACTH syndrome; p-NETs: pancreatic neuroendocrine tumors; EAS-p-NETs: EAS due to p-NETs; ACTH: adrenocorticotropic hormone; UFC: urinary free cortisol; SSA: somatostatin analog; LAR: long-acting release; SD: stable disease; PD: progressive disease; PR: partial response; N/A: not available.

## Data Availability

The data used in the study are available upon request to the corresponding author.

## Supplementary Materials

Supplementary Materials Supplement Table 1: symptoms and hormone levels of 13 patients with EAS-p-NETs. Supplement Table 2: tumor characteristics of 13 patients with EAS-p-NETs. Supplement Table 3: treatment and prognosis of 13 patients with EAS-p-NETs.
