# Supplementary material for: Somatostatin Treatment for Ectopic ACTH Syndrome due to Pancreatic Neuroendocrine Tumors: Review of the Literature
Source: Int J Endocrinol. 2022 Feb 28;2022:6283706. doi: 10.1155/2022/6283706 (PMC8901294; doi:10.1155/2022/6283706)
Supplement: Supplementary Materials — Supplement Table 1: symptoms and hormone levels of 13 patients with EAS-p-NETs. Supplement Table 2: tumor characteristics of 13 patients with EAS-p-NETs. Supplement Table 3: treatment and prognosis of 13 patients with EAS-p-NETs. [file 6283706.f1.docx]

**Supplement Table** **1** **Symptoms and hormone levels of 13 patients with EAS-p-NETs.**

| **No.** | **Year** | **First Author** | **Age at Diagnosis** | **Sex** | **Cushingoid Manifestations** | **Serum Potassium (mmol/L)** | **Diabetes Mellitus** | **Hypertension** | **Morning ACTH (pg/ml)** | **Morning Cortisol**  **(ug/dl)** | **24hUFC (ug/d)** |
| --- | --- | --- | --- | --- | --- | --- | --- | --- | --- | --- | --- |
| 1 | 2020 | our case | 23 | F | Yes | 3.3 | Yes | Yes | 139 | 24.1 | 653.64 |
| 2 | 2017 | do Amor Divino PH^[14]^ | 58 | M | Yes | N/A | Yes | Yes | 190 | 48 | 6519 |
| 3 | 2016 | Tadokoro, R^[15]^ | 61 | F | Yes | 2.6 | Yes | N/A | 205.9 | 15.2 | 86.1 |
| 4 | 2014 | Sauer N^[16]^ | 46 | M | Yes | 2.4 | Yes | Yes | 247 | 1198 | 38200 |
| 5 | 2014 | Rajeev SP^[17]^ | 34 | M | Yes | 2.5 | Yes | No | 227 | 72.6 | N/A |
| 6 | 2013 | Patel FB^[18]^ | 44 | F | Yes | Yes | Yes | Yes | N/A | N/A | N/A |
| 7 | 2010 | Kondo, T^[19]^ | 64 | F | Yes | 2.7 | Yes | Yes | 340 | 26.9 | 893 |
| 8 | 2003 | Doi, M^[20]^ | 21 | F | Yes | 2 | No | Yes | 735 | 145 | N/A |
| 9 | 1999 | Gill, GV^[21]^ | 31 | F | Yes | 2.2 | Yes | Yes | 177 | 78.2 | 2102 |
| 10 | 1993 | Woodhouse，NJ^[22]^ | 33 | F | Yes | 2.9 | N/A | N/A | 436 | 181.2 | N/A |
| 11 | 1989 | Bertagna, X^[23]^ | 37 | F | Yes | 1.7 | Yes | No | 218 | 26 | 550 |
| 12 | 1988 | Lamberts, SW^[24]^ | 37 | M | Yes | 3.8 | Yes | Yes | 68.1 | 32 | 1080 |
| 13 | 1988 | Ruszniewski，P^[25]^ | 49 | F | Yes | 2.7 | Yes | Yes | 138 | 16.5 | N/A |

EAS: ectopic ACTH syndrome; p-NETs: pancreatic neuroendocrine tumors; EAS-p-NETs: EAS due to p-NETs; ACTH: adrenocorticotropic hormone; UFC: urinary free cortisol; N/A: not available.

**Supplement Table** **2** **Tumor characteristics of 13 patients with EAS-p-NETs.**

| **No.** | **First Author** | **Pancteatic Tumor Size** | **Tumor Metastasis** | **Percentage of Ki-67** | **Positive Immunostaining of ACTH** | **Positive Immunostaining of SSTR-2** | **Positive Immunostainning of SSTR-5** | **Response to Octreotide Loading Test** |
| --- | --- | --- | --- | --- | --- | --- | --- | --- |
| 1 | our case | 1.1×0.8cm | lymph nodes and multiple liver metastasis | 10-15% | Negative | Positive | N/A | Yes |
| 2 | do Amor Divino PH^[14]^ | 5cm | multiple liver metastasis | 6% | Positive | N/A | N/A | N/A |
| 3 | Tadokoro, R^[15]^ | 6cm | lymph nodes metastasis | 11.5% | Positive | N/A | N/A | N/A |
| 4 | Sauer N^[16]^ | 1.2cm | liver metastasis | ＜1% | Positive | N/A | N/A | N/A |
| 5 | Rajeev SP^[17]^ | N/A | lymph nodes and liver metastasis | 20% | N/A | N/A | N/A | N/A |
| 6 | Patel FB^[18]^ | large | multiple liver metastasis | N/A | Negative | N/A | N/A | N/A |
| 7 | Kondo, T^[19]^ | 2.5×2.5cm | multiple liver metastasis | 10% | Positive | Positive | Positive | Yes |
| 8 | Doi, M^[20]^ | 5×3.5×2cm | liver and lymph nodes metastasis | N/A | Positive | Positive | N/A | Yes |
| 9 | Gill, GV^[21]^ | N/A | liver, bone and pelvic metastasis | N/A | N/A | N/A | N/A | No/Yes |
| 10 | Woodhouse，NJ^[22]^ | N/A | extensive liver metastasis | N/A | N/A | N/A | N/A | Yes |
| 11 | Bertagna, X^[23]^ | N/A | diffuse metestasis | N/A | N/A | N/A | N/A | Yes |
| 12 | Lamberts, SW^[24]^ | 5×3cm | lymph nodes metastasis | N/A | Positive | N/A | N/A | Yes |
| 13 | Ruszniewski，P^[25]^ | N/A | multiple liver metastases | N/A | N/A | N/A | N/A | Yes |

EAS: ectopic ACTH syndrome; p-NETs: pancreatic neuroendocrine tumors; EAS-p-NETs: EAS due to p-NETs; ACTH: adrenocorticotropic hormone; UFC: urinary free cortisol; SSTR: somatostatin receptor; N/A: not available.

**Supplement Table** **3** **Treatment and prognosis of 13 patients with EAS-p-NETs.**

| **No.** | **First Author** | **Treatment Procedure** | **Side Effects of SSA** | **Effect of Controlling Effect of ACTH and Cortisol after SSA Treatment** | **Effect of Tumor Growth after SSA Treatment** | **Duration of Follow-up** | **Prognosis** |
| --- | --- | --- | --- | --- | --- | --- | --- |
| 1 | our case | octreotide LAR 20 mg/month for 23 cycles;body-caudal pancreatectomy,splenectomy,radiofrequency ablation combined with resection of multiple hepatic metastasis | mild abdominal pain and diarrhea at first 2 weeks,gallstone | ACTH and cortison normalized for 18m | **SD**: the sizes and numbers of liver metastasis unchanged | 28 m | Scheduled to PRRT |
| 2 | do Amor Divino PH^[14]^ | body-caudal pancreatectomy, splenectomy and removal of the segment III of the liver;10m later octreotide LAR 20 mg IM once every 28 days;hepatic embolisation;bilateral adrenalectomy;oxaliplatin and capecitabine | N/A | unsuccessful in controlling symptoms | **PD:**disease progression after two doses | 42 m | N/A |
| 3 | Tadokoro, R^[15]^ | octreotide LAR 20 mg/month for 36m and metyrapone | cholecystitis and peritonitis | ACTH and cortisol level increased during octreotide LAR treatment | **PD**:the tumors gradually increased in size | 36 m | died of tumor after 36 months of octreotide LAR therapy |
| 4 | Sauer N^[16]^ | ketoconazole and emergency bilateral adrenalectomy;octreotide for 2w,distal splenopancreatectomy and right hemihepatectomy | N/A | N/A | N/A | N/A | N/A |
| 5 | Rajeev SP^[17]^ | Whipple’s procedure,Lanreotide and Sunitinib for 2y | N/A | ACTH and cortisol increased | **PD**:tumor progression | 27 m | Chemotherapy lowered ACTH and cortisol, reduced the size of the hepatic metastases.The patient remained clinically stable three months later |
| 6 | Patel FB^[18]^ | ketoconazole and metayrapone followed by open distal pancreatectomy and splenectomy; 5.5 years later recurrence as liver metastasis; octreotide LAR 20 mg/month for 2m and Y90 radioembolization | no side effects | ACTH and UFC levels normalized | N/A | 74 m | N/A |
| 7 | Kondo, T^[19]^ | octreotide LAR 20 mg/month(15 times) along with metyrapone 750mg/d(3 months), followed by transarterial chemoembolization(10 times) | N/A | combination of these treatments effectively decreased ACTH and cortisol levels in 3 months | **PR**:liver metastasis reduced in number and size and pancreatic tumor unchanged for more than 20 months | 20 m | plan to radical surgical treatment |
| 8 | Doi, M^[20]^ | intermittent octreotide 100-300ug/d followed metyrapone, chemoembolization and hepatectomy liver transplantation | N/A | intermittent usage of octeotide combined with other therapies showed long-term effectiveness to control of ACTH hypersecretion | **PD:**fail to decrease tumor growth | 61 m | died of ARDS after 5 years of intensive multidisciplinary treatment |
| 9 | Gill, GV^[21]^ | high doses of octreotide(500ug tid), metyrapone(1g tid) and ketoconazole(200mg tid), Hepatic polystyrene embolisation and liver transplantation | N/A | high doses of octreotide failed before liver transplantation. After tumor recurred,high doses of octreotide normalise her biochemically | **PD**: tumor grew rapidly | 18 m | died of tumor soon after 10 months post-transplate |
| 10 | Woodhouse，NJ^[22]^ | bilateral adrenalectomy and octeotide increased progressively from 100 to 500 ug every 8 hours for 18 months | no side effects | a rapid and sustained reduction in ACTH levels was observed | **PR**:a reduction in the size of the liver metastases was seen on CT scans | 18 m | N/A |
| 11 | Bertagna, X^[23]^ | 5-fluorouracil,cis-platinum and streptozotocin and octeotide 100ug tid | bowel movements increased and fatty stools | decreased to normal within 3 days after the initiation of octreotide therapy (150, 300, and 600 micrograms/day).Urinary cortisol excretion remained normal for 2 months during chronic octreotide therapy.hypercortisolism returned 2m later | **PD**:tumor progressed | 4 m | died of hepatic insufficiency 4 months after octertide treatment |
| 12 | Lamberts, SW^[24]^ | octreotide (100 ug/d tid) | several small gallstones within gallbladder | normalized plasma ACTH and cortisol levels and urinary cortisol excretion | **PR**:tumor growth deminished after 6 months of SMS 201No995 therapy | 6 m | N/A |
| 13 | Ruszniewski，P^[25]^ | splenopancreatectomy and total gastrectomy and hepatic metastases removed,chemotherapy with Streptozotocin and 5 Fluorouracil, octreotide at a dosage of 50μg sc | no side effects | decreased to normal | **SD**:size and number of liver metastases slightly increased at 9 months | 80 m | N/A |

EAS: ectopic ACTH syndrome; p-NETs: pancreatic neuroendocrine tumors; EAS-p-NETs: EAS due to p-NETs; ACTH: adrenocorticotropic hormone; UFC: urinary free cortisol; SSA: somatostatin analog; LAR: long-acting release; SD: stable disease; PD: progressive disease; PR: partial response; PRRT: peptide receptor radionuclide therapy; N/A: not available.
